# Supplementary material for: Association of total cholesterol variability with risk of venous thromboembolism: A nationwide cohort study
Source: PLoS One. 2023 Aug 17;18(8):e0289743. doi: 10.1371/journal.pone.0289743 (PMC10434969; doi:10.1371/journal.pone.0289743)
Supplement: S1 Method — (DOCX) [file pone.0289743.s001.docx]

**Supplemental method 1. Information about data source**

The National Health Insurance Service (NHIS) is the only provider of insurance in Korea, controlled and supported by the Korean government, and nearly 97% of the population is covered under its services, while the Medical Aid program, run by the NHIS supports the remaining 3% of individuals. NHIS subscribers are encouraged to undergo yearly health screening. The National Health Insurance Service-National Health Screening (NHIS-HEALS) cohort used in our study comprises of approximately 1,236,589 individuals aged between 40 to 79 years, who attended at least three annual health screening between 2003 and 2008. These individuals were selected through a process of stratified random sampling. The selected individuals represented approximately 10% of the overall population that underwent a national health examination with the dataset number being NIHS-2022-01-313.The NHIS-HEALS cohort database contains individual’s demographic, socioeconomic status, and health screening data. Additinally, the claims database comprises information on diagnosis, prescription and treatment modality. The health screening conducted on participants in the NHIS-HEALS cohort included measurements of heights, weight, blood pressure, laboratory test, and response to questionnaire on life style such as smoking and alcohol consumption history. However, the participants themselves were not involved in the design, analysis, or reporting of this study. This study received approval from the Institutional Review Board (IRB) of Ewha Womans University College of Medicine (SEUMC 2022-02-018). The IRB granted permission for the analysis to be conducted and waived the requirement of consent.
