# Supplementary material for: Association of total cholesterol variability with risk of venous thromboembolism: A nationwide cohort study
Source: PLoS One. 2023 Aug 17;18(8):e0289743. doi: 10.1371/journal.pone.0289743 (PMC10434969; doi:10.1371/journal.pone.0289743)
Supplement: S1 Table — (DOCX) [file pone.0289743.s002.docx]

**S1 Table.** Definitions of covariates

| Diagnosis | ICD-10 code | Diagnostic definition |
| --- | --- | --- |
| Hypertension | At least one claim for I10-15 with the prescription of antihypertensive agents. | Systolic/diastolic blood pressure ≥ 140/90mmHg at baseline health examination or self-reported hypertension in the questionnaire. |
| Diabetes mellitus | At least one claim for E11-14ith the prescription of antidiabetic agents or two or more claims for E11-14. | Fasting glucose level ≥ 7.0 mmol/L at baseline health examination or self-reported diabetes mellitus in questionnaire. |
| Dyslipidemia | E78 with the prescription of with prescription of dyslipidemia-related agents including statin or two or more claims for E78 | Total cholesterol ≥ 240 mg/dL or low-density lipoprotein > 160 mg/dL or high-density lipoprotein ≥ 4o mg/dL or prescription of dyslipidemia-related agents |
| Atrial fibrillation | Two or more claims for the I48. |  |
| Cancer | C00-97 with a specific registration code of V027 or V193-4 |  |
| Renal disease | Two or more claims for N17-19, I12-13, E082, E102, E112, E132 | An estimated glomerular filtration rate < 60mL/min/1.73m^2^ |
| Antiphospholipid syndrome | Two or more claims for D68.6 and specific registration code of V253 for a rare intractable disease program |  |
| Osteoporotic fracture | S22.0, S22.1, S32.0, S32.7, T08, M48.4, M48.5, M49.5 for vertebral fracture or S72.0, S72.1 for hip fracture or S52.5, S52.6 for distal radius fracture or S42.2, S42.3 for humerus fracture. |  |
