# Supplementary material for: Association of total cholesterol variability with risk of venous thromboembolism: A nationwide cohort study
Source: PLoS One. 2023 Aug 17;18(8):e0289743. doi: 10.1371/journal.pone.0289743 (PMC10434969; doi:10.1371/journal.pone.0289743)
Supplement: S3 Table — (DOCX) [file pone.0289743.s004.docx]

**S3 Table.** The risk for the occurrence of venous thromboembolism according to the quartiles of total cholesterol variability in women

|  |  |  |  |  |  | Multivariable model (1) |  |  | Multivariable model (2) |  |  |
| --- | --- | --- | --- | --- | --- | --- | --- | --- | --- | --- | --- |
|  | Number of  participants | Number of  events | Event rate (%) (95% CI) | Person-years | Incidence rate (per 1000 person-years) | Adjusted HR (95% CI) | *P*-value | *P*-value for trend | Adjusted HR (95% CI) | *P*-value | *P*-value for trend |
| CV |  |  |  |  |  |  |  | <.001 |  |  | <.001 |
| Q1 | 62294 | 725 | 1.16  (1.08, 1.25) | 762786.58 | 0.95 | 1 (reference) |  |  | 1 (reference) |  |  |
| Q2 | 62295 | 678 | 1.09  (1.01, 1.17) | 764122.74 | 0.89 | 0.98  (0.88, 1.09) | 0.707 |  | 1.00  (0.90, 1.11) | 0.998 |  |
| Q3 | 62295 | 739 | 1.19  (1.1, 1.27) | 763558.28 | 0.97 | 1.02  (0.92,1.13) | 0.742 |  | 1.03  (0.95, 1.12) | 0.307 |  |
| Q4 | 62295 | 927 | 1.49  (1.39, 1.58) | 759651.75 | 1.22 | 1.06  (1.01, 1.11) | 0.002 |  | 1.06  (1.01, 1.12) | 0.004 |  |
| SD |  |  |  |  |  |  |  | <.001 |  |  | <.001 |
| Q1 | 62292 | 610 | 0.98  (0.9, 1.06) | 763727.77 | 0.8 | 1 (reference) |  |  | 1 (reference) |  |  |
| Q2 | 62287 | 685 | 1.1  (1.02, 1.18) | 764380.39 | 0.9 | 1.03  (0.97, 1.09) | 0.162 |  | 1.03  (0.98, 1.08) | 0.157 |  |
| Q3 | 62301 | 757 | 1.22  (1.13, 1.3) | 763197.89 | 0.99 | 1.06  (0.95, 1.18) | 0.308 |  | 1.05  (0.94, 1.17) | 0.384 |  |
| Q4 | 62299 | 1017 | 1.63  (1.53, 1.73) | 758813.3 | 1.34 | 1.07  (1.01, 1.13) | 0.003 |  | 1.07  (1.01, 1.12) | 0.004 |  |
| VIM |  |  | #DIV/0! |  |  |  |  | <.001 |  |  | <.001 |
| Q1 | 62294 | 610 | 0.98  (0.9, 1.06) | 763752.48 | 0.80 | 1 (reference) |  |  | 1 (reference) |  |  |
| Q2 | 62295 | 685 | 1.1  (1.02, 1.18) | 764478.94 | 0.90 | 1.03  (0.97, 1.09) | 0.163 |  | 1.04  (0.97, 1.10) | 0.196 |  |
| Q3 | 62295 | 757 | 1.22  (1.13, 1.3) | 763124.06 | 0.99 | 1.06  (0.95, 1.18) | 0.305 |  | 1.05  (0.94, 1.17) | 0.378 |  |
| Q4 | 62295 | 1017 | 1.63  (1.53, 1.73) | 758763.88 | 1.34 | 1.08  (1.01, 1.15) | 0.009 |  | 1.07  (1.01, 1.12) | 0.012 |  |

Multivariable model (1) was adjusted for age, sex, body mass index, household income levels, smoking, alcohol consumption, regular physical activity, hypertension, diabetes mellitus, dyslipidemia, stroke, atrial fibrillation, renal disease, cancer, and on lipid-lowering agent.

Multivariable model (2) was adjusted for age, sex, body mass index, household income levels, smoking, alcohol consumption, regular physical activity, hypertension, diabetes mellitus, dyslipidemia, stroke, atrial fibrillation, renal disease, cancer, on lipid-lowering agent, and mean TC.

HR, hazard ratio; CI, confidence interval; CV, coefficient of variation; Q, quartile; SD, standard deviation; VIM, variability independent of the mean
