# Supplementary material for: Association of total cholesterol variability with risk of venous thromboembolism: A nationwide cohort study
Source: PLoS One. 2023 Aug 17;18(8):e0289743. doi: 10.1371/journal.pone.0289743 (PMC10434969; doi:10.1371/journal.pone.0289743)
Supplement: S4 Table — (DOCX) [file pone.0289743.s005.docx]

**S4 Table.** The risk of occurrence of venous thromboembolism according to quartiles of total cholesterol variability additionally adjusting the use of lipid lowering agents and mean total cholesterol levels.

|  |  |  | Multivariable model (1) |  | Multivariable model (2) |  |
| --- | --- | --- | --- | --- | --- | --- |
| Variable | Crude HR (95% CI) | p-value | Adjusted HR (95% CI) | p-value | Adjusted HR (95% CI) | p-value |
| Age, years | 1.08 (1.07, 1.08) | <.001 | 1.06 (1.06, 1.07) | <.001 | 1.06 (1.06, 1.07) | <.001 |
| Sex |  |  |  |  |  |  |
| Male | 1 (reference) |  | 1 (reference) |  | 1 (reference) |  |
| Female | 1.17 (1.13, 1.22) | <.001 | 1.00 (0.95, 1.05) | 0.947 | 1.00 (0.96, 1.06) | 0.875 |
| Body mass index (kg/m2) | 1.07 (1.06, 1.07) | <.001 | 1.05 (1.04, 1.05) | <.001 | 1.05 (1.04, 1.05) | <.001 |
| Household income |  |  |  |  |  |  |
| Q1, lowest | 1 (reference) |  | 1 (reference) |  | 1 (reference) |  |
| Q2 | 0.60 (0.57, 0.63) | <.001 | 1.00 (0.95, 1.06) | 0.891 | 1.00 (0.95, 1.06) | 0.905 |
| Q3 | 0.49 (0.47, 0.52) | <.001 | 0.89 (0.84, 0.94) | <.001 | 0.89 (0.84, 0.94) | <.001 |
| Q4, highest | 0.52 (0.49, 0.55) | <.001 | 0.72 (0.68, 0.76) | <.001 | 0.72 (0.68, 0.76) | <.001 |
| Smoking status |  |  |  |  |  |  |
| Never | 1 (reference) |  | 1 (reference) |  | 1 (reference) |  |
| Former | 0.87 (0.82, 0.92) | <.001 | 1.01 (0.95, 1.07) | 0.848 | 1.01 (0.95, 1.07) | 0.826 |
| Current | 0.77 (0.74, 0.81) | <.001 | 1.10 (1.05, 1.16) | <.001 | 1.10 (1.05, 1.16) | <.001 |
| Alcohol consumption (days/week) |  |  |  |  |  |  |
| None | 1 (reference) |  | 1 (reference) |  | 1 (reference) |  |
| 1-4 | 0.82 (0.79, 0.86) | <.001 | 0.97 (0.93, 1.01) | 0.120 | 0.97 (0.93, 1.01) | 0.125 |
| ≥ 5 | 1.80 (1.62, 1.99) | <.001 | 1.23 (1.11, 1.37) | <.001 | 1.23 (1.11, 1.37) | <.001 |
| Regular physical activity (days/week) |  |  |  |  |  |  |
| None | 1 (reference) |  | 1 (reference) |  | 1 (reference) |  |
| 1-4 | 0.88 (0.85, 0.92) | <.001 | 0.90 (0.86, 0.93) | <.001 | 0.90 (0.86, 0.93) | <.001 |
| ≥ 5 | 1.35 (1.26, 1.44) | <.001 | 0.98 (0.92, 1.05) | 0.627 | 0.98 (0.92, 1.05) | 0.614 |
| Comorbidities |  |  |  |  |  |  |
| Hypertension | 2.90 (2.79, 3.00) | <.001 | 1.38 (1.32, 1.44) | <.001 | 1.37 (1.32, 1.43) | <.001 |
| Diabetes mellitus | 2.51 (2.40, 2.63) | <.001 | 1.19 (1.14, 1.25) | <.001 | 1.19 (1.13, 1.25) | <.001 |
| Dyslipidemia | 2.07 (1.99, 2.15) | <.001 | 1.15 (1.10, 1.20) | <.001 | 1.17 (1.12, 1.22) | <.001 |
| Stroke | 3.72 (3.37, 4.11) | <.001 | 1.22 (1.10, 1.35) | <.001 | 1.21 (1.10, 1.34) | <.001 |
| Atrial fibrillation | 4.71 (4.10, 5.42) | <.001 | 1.91 (1.66, 2.20) | <.001 | 1.90 (1.65, 2.19) | <.001 |
| Renal disease | 3.32 (3.02, 3.66) | <.001 | 1.34 (1.22, 1.48) | <.001 | 1.33 (1.21, 1.47) | <.001 |
| Cancer | 2.78 (2.58, 3.01) | <.001 | 1.58 (1.46, 1.71) | <.001 | 1.58 (1.46, 1.71) | <.001 |
| Antiphospholipid syndrome | 2.65 (2.19, 3.20) | <.001 | 1.42 (1.17, 1.71) | <.001 | 1.41 (1.17, 1.71) | <.001 |
| Osteoporotic fracture | 1.83 (1.64, 2.06) | <.001 | 1.21 (0.92, 1.52) | 0.169 | 1.20 (0.82, 1.04) | 0.168 |
| On lipid-lowering agent | 0.89 (0.82, 0.96) | <.001 | 0.92 (0.83, 1.04) | 0.277 | 0.91 (0.82, 1.02) | 0.152 |
| Mean total cholesterol (mg/dL) | 1.02 (1.01, 1.03) | <.001 |  |  | 1.02 (1.00, 1.02) | 0.056 |
| Total cholesterol variability with CV |  |  |  |  |  |  |
| Q1 | 1 (reference) |  | 1 (reference) |  | 1 (reference) |  |
| Q2 | 1.02 (0.96, 1.07) | 0.594 | 1.05 (0.99, 1.11) | 0.086 | 1.05 (0.99, 1.11) | 0.092 |
| Q3 | 1.14 (1.08, 1.20) | <.001 | 1.12 (1.06, 1.18) | <.001 | 1.12 (1.06, 1.18) | <.001 |
| Q4 | 1.48 (1.41, 1.55) | <.001 | 1.14 (1.08, 1.20) | <.001 | 1.14 (1.08, 1.20) | <.001 |

Multivariable model (1) was adjusted for sex, age, body mass index, household income levels, smoking, alcohol consumption, regular physical activity, hypertension, diabetes mellitus, dyslipidemia, stroke, atrial fibrillation, renal disease, cancer, antiphospholipid syndrome, and osteoporotic fracture and on lipid-lowering agent.

Multivariable model (2) was adjusted for sex, age, body mass index, household income levels, smoking, alcohol consumption, regular physical activity, hypertension, diabetes mellitus, dyslipidemia, stroke, atrial fibrillation, renal disease, cancer, antiphospholipid syndrome, osteoporotic fracture, on lipid-lowering agent, and mean total cholesterol.

HR, hazard ratio, CI, confidence interval, CV, coefficient of variation; Q, Quartile; SD, standard deviation; VIM, variability independent of the mean.
