# Supplementary material for: Association of total cholesterol variability with risk of venous thromboembolism: A nationwide cohort study
Source: PLoS One. 2023 Aug 17;18(8):e0289743. doi: 10.1371/journal.pone.0289743 (PMC10434969; doi:10.1371/journal.pone.0289743)
Supplement: S5 Table — (DOCX) [file pone.0289743.s006.docx]

**S5 Table.** The risk for occurrence of venous thromboembolism according to deciles of total cholesterol variability

|  |  |  |  |  |  | Multivariable model (1) |  |  | Multivariable model (2) |  |  |
| --- | --- | --- | --- | --- | --- | --- | --- | --- | --- | --- | --- |
|  | Number of  participants | Number of  events | Event rate (%) (95% CI) | Person-years | Incidence rate (per 1000 person-years) | Adjusted HR (95% CI) | p-value | p-value for trend | Adjusted HR (95% CI) | p-value | p-value for trend |
| CV |  |  |  |  |  |  |  | <.001 |  |  | <.001 |
| D1 | 108502 | 1029 | 0.95 (0.89, 1.01) | 1324463.65 | 0.76 | 1 (reference) |  |  | 1 (reference) |  |  |
| D2 | 108502 | 997 | 0.92 (0.86, 0.98) | 1327584.76 | 0.74 | 1.06 (0.97, 1.15) | 0.211 |  | 1.06 (0.97, 1.15) | 0.211 |  |
| D3 | 108503 | 1047 | 0.96 (0.91, 1.02) | 1327221.70 | 0.78 | 1.12 (1.03, 1.22) | 0.009 |  | 1.12 (1.03, 1.22) | 0.009 |  |
| D4 | 108502 | 1042 | 0.96 (0.90, 1.02) | 1327151.77 | 0.77 | 1.10 (1.01, 1.20) | 0.024 |  | 1.10 (1.01, 1.20) | 0.025 |  |
| D5 | 108503 | 1018 | 0.94 (0.88, 1.00) | 1326955.56 | 0.76 | 1.07 (0.98, 1.17) | 0.120 |  | 1.07 (0.98, 1.17) | 0.125 |  |
| D6 | 108502 | 1134 | 1.05 (0.98, 1.11) | 1325983.95 | 0.84 | 1.17 (1.08, 1.27) | <.001 |  | 1.17 (1.08, 1.27) | <.001 |  |
| D7 | 108503 | 1114 | 1.03 (0.97, 1.09) | 1324715.53 | 0.83 | 1.13 (1.03, 1.23) | 0.006 |  | 1.12 (1.03, 1.22) | 0.007 |  |
| D8 | 108502 | 1237 | 1.14 (1.08, 1.20) | 1322562.49 | 0.92 | 1.19 (1.10, 1.30) | <.001 |  | 1.19 (1.10, 1.29) | <.001 |  |
| D9 | 108503 | 1345 | 1.24 (1.17, 1.31) | 1318141.81 | 1.01 | 1.18 (1.09, 1.28) | <.001 |  | 1.18 (1.09, 1.28) | <.001 |  |
| D10 | 108502 | 1805 | 1.66 (1.59, 1.74) | 1304350.12 | 1.37 | 1.22 (1.13, 1.32) | <.001 |  | 1.21 (1.12, 1.31) | <.001 |  |
| SD |  |  |  |  |  |  |  | 0.001 |  |  | <.001 |
| D1 | 108530 | 970 | 0.89 (0.84, 0.95) | 1325788.59 | 0.72 | 1 (reference) |  |  | 1 (reference) |  |  |
| D2 | 108514 | 928 | 0.86 (0.80, 0.91) | 1328209.35 | 0.69 | 1.02 (0.94, 1.12) | 0.610 |  | 1.03 (0.94, 1.12) | 0.584 |  |
| D3 | 108420 | 974 | 0.90 (0.84, 0.95) | 1326878.81 | 0.72 | 1.05 (0.96, 1.15) | 0.254 |  | 1.06 (0.97, 1.16) | 0.221 |  |
| D4 | 108537 | 1010 | 0.93 (0.87, 0.99) | 1327838.30 | 0.75 | 1.08 (0.99, 1.18) | 0.096 |  | 1.08 (0.99, 1.18) | 0.074 |  |
| D5 | 108490 | 1069 | 0.99 (0.93, 1.04) | 1325670.48 | 0.80 | 1.11 (1.02, 1.21) | 0.018 |  | 1.12 (1.03, 1.22) | 0.012 |  |
| D6 | 108551 | 1112 | 1.02 (0.96, 1.08) | 1326771.08 | 0.83 | 1.12 (1.03, 1.22) | 0.010 |  | 1.13 (1.04, 1.23) | 0.005 |  |
| D7 | 108452 | 1143 | 1.05 (0.99, 1.12) | 1323610.65 | 0.85 | 1.10 (1.01, 1.20) | 0.029 |  | 1.11 (1.02, 1.21) | 0.015 |  |
| D8 | 108535 | 1301 | 1.20 (1.13, 1.26) | 1321429.14 | 0.97 | 1.18 (1.09, 1.29) | <.001 |  | 1.20 (1.10, 1.30) | <.001 |  |
| D9 | 108481 | 1407 | 1.30 (1.23, 1.36) | 1317267.83 | 1.05 | 1.15 (1.06, 1.25) | 0.001 |  | 1.17 (1.08, 1.27) | <.001 |  |
| D10 | 108514 | 1854 | 1.71 (1.63, 1.79) | 1305667.09 | 1.40 | 1.16 (1.07, 1.25) | <.001 |  | 1.18 (1.09, 1.28) | <.001 |  |
| VIM |  |  |  |  |  |  |  | 0.001 |  |  | <.001 |
| D1 | 108502 | 969 | 0.89 (0.84, 0.95) | 1325451.17 | 0.72 | 1 (reference) |  |  | 1 (reference) |  |  |
| D2 | 108502 | 929 | 0.86 (0.80, 0.91) | 1328050.45 | 0.69 | 1.03 (0.94, 1.12) | 0.584 |  | 1.03 (0.94, 1.12) | 0.559 |  |
| D3 | 108503 | 974 | 0.90 (0.84, 0.95) | 1327884.30 | 0.72 | 1.05 (0.96, 1.15) | 0.255 |  | 1.06 (0.97, 1.16) | 0.222 |  |
| D4 | 108502 | 1010 | 0.93 (0.87, 0.99) | 1327418.74 | 0.75 | 1.08 (0.99, 1.18) | 0.090 |  | 1.09 (0.99, 1.19) | 0.070 |  |
| D5 | 108503 | 1069 | 0.99 (0.93, 1.04) | 1325842.37 | 0.80 | 1.11 (1.02, 1.21) | 0.018 |  | 1.12 (1.03, 1.22) | 0.012 |  |
| D6 | 108502 | 1112 | 1.02 (0.96, 1.09) | 1326161.36 | 0.83 | 1.12 (1.03, 1.22) | 0.009 |  | 1.13 (1.04, 1.24) | 0.005 |  |
| D7 | 108503 | 1143 | 1.05 (0.99, 1.11) | 1324241.47 | 0.85 | 1.10 (1.01, 1.20) | 0.029 |  | 1.11 (1.02, 1.21) | 0.016 |  |
| D8 | 108502 | 1301 | 1.20 (1.13, 1.26) | 1321020.49 | 0.97 | 1.19 (1.09, 1.29) | <.001 |  | 1.20 (1.10, 1.31) | <.001 |  |
| D9 | 108503 | 1407 | 1.30 (1.23, 1.36) | 1317542.96 | 1.05 | 1.15 (1.06, 1.25) | 0.001 |  | 1.17 (1.08, 1.27) | <.001 |  |
| D10 | 108502 | 1854 | 1.71 (1.63, 1.79) | 1305518.02 | 1.41 | 1.16 (1.07, 1.26) | <.001 |  | 1.18 (1.09, 1.28) | <.001 |  |

Multivariable model (1) was adjusted for sex, age, body mass index, household income levels, smoking, alcohol consumption, regular physical activity, hypertension, diabetes mellitus, dyslipidemia, stroke, atrial fibrillation, renal disease, cancer, antiphospholipid syndrome, osteoporotic fracture and on lipid-lowering agent..

Multivariable model (2) was adjusted for sex, age, body mass index, income levels, smoking, alcohol consumption, regular physical activity, hypertension, diabetes mellitus, dyslipidemia, stroke, atrial fibrillation, renal disease, cancer, antiphospholipid syndrome, osteoporotic fracture, on lipid-lowering agent, and mean total cholesterol.

CI, confidence interval; HR, hazard ratio, CV, coefficient of variation; Q, Quartile; SD, standard deviation; VIM, variability independent of the mean.
