# Supplementary material for: Association of total cholesterol variability with risk of venous thromboembolism: A nationwide cohort study
Source: PLoS One. 2023 Aug 17;18(8):e0289743. doi: 10.1371/journal.pone.0289743 (PMC10434969; doi:10.1371/journal.pone.0289743)
Supplement: S9 Table — (DOCX) [file pone.0289743.s010.docx]

**S9 Table.** The risk of occurrence of pulmonary embolism according to quartiles of total cholesterol variability

|  | Number of  participants | Number  of events | Event rate (%) (95% CI) | Person-years | Incidence rate (per 1000 person-years) | Adjusted HR (95% CI) | p-value | p-value for trend |
| --- | --- | --- | --- | --- | --- | --- | --- | --- |
| CV |  |  |  |  |  |  |  | 0.046 |
| Q1 | 271256 | 707 | 0.26 (0.24, 0.28) | 3323400.17 | 0.21 | 1 (reference) |  |  |
| Q2 | 271256 | 696 | 0.26 (0.24, 0.28) | 3326172.09 | 0.21 | 1.03 (0.93, 1.14) | 0.576 |  |
| Q3 | 271256 | 751 | 0.28 (0.26, 0.30) | 3321334.75 | 0.23 | 1.07 (0.96, 1.18) | 0.214 |  |
| Q4 | 271256 | 955 | 0.35 (0.33, 0.37) | 3295485.61 | 0.29 | 1.09 (1.01, 1.17) | 0.050 |  |
| SD |  |  |  |  |  |  |  | 0.025 |
| Q1 | 271272 | 682 | 0.25 (0.23, 0.27) | 3325034.51 | 0.21 | 1 (reference) |  |  |
| Q2 | 271219 | 657 | 0.24 (0.22, 0.26) | 3324942.23 | 0.20 | 0.96 (0.86, 1.07) | 0.433 |  |
| Q3 | 271288 | 790 | 0.29 (0.27, 0.31) | 3320169.26 | 0.24 | 1.06 (0.96, 1.18) | 0.237 |  |
| Q4 | 271245 | 980 | 0.36 (0.34, 0.38) | 3296246.63 | 0.30 | 1.08 (1.01, 1.15) | 0.036 |  |
| VIM |  |  |  |  |  |  |  | 0.024 |
| Q1 | 271256 | 682 | 0.25 (0.23, 0.27) | 3324837.67 | 0.21 | 1 (reference) |  |  |
| Q2 | 271256 | 657 | 0.24 (0.22, 0.26) | 3325400.57 | 0.20 | 0.96 (0.86, 1.07) | 0.430 |  |
| Q3 | 271256 | 790 | 0.29 (0.27, 0.31) | 3319782.13 | 0.24 | 1.06 (0.96, 1.18) | 0.235 |  |
| Q4 | 271256 | 980 | 0.36 (0.34, 0.38) | 3296372.26 | 0.30 | 1.07 (1.011, 1.12) | 0.037 |  |

Model was adjusted for sex, age, body mass index, household income levels, smoking, alcohol consumption, regular physical activity, hypertension, diabetes mellitus, dyslipidemia, stroke, atrial fibrillation, renal disease, cancer, antiphospholipid syndrome, osteoporotic fracture, on lipid-lowering agent, and mean total cholesterol.

CI, confidence interval; HR, hazard ratio, CV, coefficient of variation; Q, Quartile; SD, standard deviation; VIM, variability independent of the mean.
