# Supplementary material for: Association of total cholesterol variability with risk of venous thromboembolism: A nationwide cohort study
Source: PLoS One. 2023 Aug 17;18(8):e0289743. doi: 10.1371/journal.pone.0289743 (PMC10434969; doi:10.1371/journal.pone.0289743)
Supplement: Attachment — Submitted filename: Respons to reviewers.docx [file pone.0289743.s013.docx]

**Answer to comments**

**MS ID:** PONE-D-22-2922R1

**MS title:** Association of total cholesterol variability with risk of venous thrombosis: a nationwide cohort study

We wish to thank the reviewer for his/her helpful and constructive review of our manuscript. We are very grateful for suggestion and insightful comments

**Reviewer #1**

**Comment)** The age of the target population ranged from 40 to 79 years (line 90). How do the authors explain that the mean age of the studied population was only 43.8 ± 10.1? The age distribution looks very close to the lower end of the targeted population and the results may only apply to a younger population as compared with older, which is at high risk for both VTE and high TC.

**Answer)** Thank the reviewer’s valuable comment. We used the Korean National Health Insurance Service-National Health Screening (NHIS-HEALS) cohort database. The NHIS-HEALS cohort is a cohort of participants who participated in health screening programs provided by the NHIS in the South Korea. This cohort was designated to offer relevant and useful data for a wide range of health researchers. This HEALS program has been applied at least once every 2 years for the entire population of Korean adults aged 40 years or older, but the NHIS-HEALS cohort was limited to adults aged 40 to 79 years because the response rate was very low among people aged 80 years or older. Therefore, the age of our target population in our study ranged from 40 to 79 years. Nevertheless, as the pointed out by the reviewer, the mean age of population included in our study was quite young (43.8 ± 10.1). As a result of skew deviation toward relatively lower margin of the age of our target population could be originated from two reasons. The first reason is that the low participation rate in older age.

The figure above illustrates the participation rate according to age group from 2002 to 2013 and the participation rate decreased as age increased. The total participation rate over aged 60 was only 25.8%. The majority of included participants were less than aged 60. Thus, low portion of participants more than aged 60 could influenced the skew deviation toward relatively lower age margin of target population. Second, according to inclusion criteria, for calculating total cholesterol variability, we excluded the participants (n=55,920) with less than 3 repeated measurements for total cholesterol. The percentage of frequency less than 3 of biennial screening participation in 2002-2013 was 14.6%. Considering the decrease in participation rates as age increases, a significant proportion of these individuals could be likely to be elderly and excluded. Therefore, it is highly possible that these factors (low participation rates in elderly and the possibility of excluding the participants who were elderly) contributed to the skew deviation of lower margin of age in target population. We added this weakness of our study in limitation with reference who previously addressed this weakness point of NHIS-HEALS cohort by Korea NHIS.

Page 15 line 281-283

Discussion

*“Finally, the mean age of target population was skewed toward lower age margin of target population. This could be originated from the lower participation rates for health screening program in elderly [27]”*

27.	Seong SS, Kim Y-Y, Park SK, Khang YH, Kim HC, Park JH, et al. Cohort profile: the National Health Insurance Service-National Health Screening Cohort (NHIS-HEALS) in Korea. BMJ open 2017;7:e016640. doi:10.1136/bmjopen-2017-016640.

**Comment)** Have the authors considered separating analysis according to sex (as sex is an important risk factor for cardiovascular disease)?

**Answer)** We performed the additional analysis according to gender. As a result, total cholesterol variability was significantly associated with the development of VT regardless of sex. We added these results in supplemental table 2 and 3 in revised manuscript.

Page 11 line 189

RESULTS

*“These findings were consistent regardless of sex (S2 and 3 Table)*

Supplementary Table 2. The risk for the occurrence of Venous thromboembolism according to the quartiles of total cholesterol variability in men

|  |  |  |  |  |  | Multivariable model (1) |  |  | Multivariable model (2) |  |  |
| --- | --- | --- | --- | --- | --- | --- | --- | --- | --- | --- | --- |
|  | Number of  participants | Number of  events | Event rate (%) (95% CI) | Person-years | Incidence rate (per 1000 person-years) | Adjusted HR (95% CI) | *P*-value | *P*-value for trend | Adjusted HR (95% CI) | *P*-value | *P*-value for trend |
| CV |  |  |  |  |  |  |  | <.001 |  |  | <.001 |
| Q1 | 208961 | 1845 | 0.88  (0.84, 0.92) | 2553101.75 | 0.72 | 1 (reference) |  |  | 1 (reference) |  |  |
| Q2 | 208961 | 1930 | 0.92  (0.88, 0.96) | 2553758.01 | 0.76 | 1.07  (1.01, 1.14) | 0.035 |  | 1.06  (1.00, 1.13) | 0.057 |  |
| Q3 | 208962 | 2117 | 1.01  (0.97, 1.06) | 2549293.55 | 0.83 | 1.13  (1.06, 1.20) | < .001 |  | 1.14  (1.07, 1.21) | < .001 |  |
| Q4 | 208961 | 2807 | 1.34  (1.29, 1.39) | 2522858.66 | 1.11 | 1.66  (1.10, 1.24) | < .001 |  | 1.17  (1.10, 1.24) | < .001 |  |
| SD |  |  | ! |  |  |  |  | <.001 |  |  | <.001 |
| Q1 | 208903 | 1767 | 0.85  (0.81, 0.89) | 2553276.21 | 0.69 | 1 (reference) |  |  | 1 (reference) |  |  |
| Q2 | 208991 | 1902 | 0.91  (0.87, 0.95) | 2553248.07 | 0.74 | 1.06  (1.00, 1.13) | 0.072 |  | 1.07  (1.00, 1.14) | 0.054 |  |
| Q3 | 208986 | 2148 | 1.03  (0.98, 1.07) | 2548333.46 | 0.84 | 1.12  (1.05, 1.20) | < .001 |  | 1.14  (1.07, 1.21) | < .001 |  |
| Q4 | 208965 | 2882 | 1.38  (1.33, 1.43) | 2524154.23 | 1.14 | 1.16  (1.09, 1.23) | < .001 |  | 1.17  (1.09, 1.24) | < .001 |  |
| VIM |  |  |  |  |  |  |  | <.001 |  |  | <.001 |
| Q1 | 208961 | 1768 | 0.85  (0.81, 0.89) | 2553985.08 | 0.69 | 1 (reference) |  |  | 1 (reference) |  |  |
| Q2 | 208961 | 1901 | 0.91  (0.87, 0.95) | 2552884.49 | 0.74 | 1.06  (0.99, 1.13) | 0.075 |  | 1.07  (1.00, 1.14) | 0.055 |  |
| Q3 | 208962 | 2148 | 1.03  (0.98, 1.07) | 2548037.39 | 0.84 | 1.12  (1.05, 1.19) | < .001 |  | 1.14  (1.07, 1.21) | < .001 |  |
| Q4 | 208961 | 2882 | 1.38  (1.33, 1.43) | 2524105.01 | 1.14 | 1.16  (1.09) | < .001 |  | 1.17  (1.09, 1.24) | < .001 |  |

Multivariable model (1) was adjusted for age, sex, body mass index, household income levels, smoking, alcohol consumption, regular physical activity, hypertension, diabetes mellitus, dyslipidemia, stroke, atrial fibrillation, renal disease, cancer, and on lipid-lowering agent.

Multivariable model (2) was adjusted for age, sex, body mass index, household income levels, smoking, alcohol consumption, regular physical activity, hypertension, diabetes mellitus, dyslipidemia, stroke, atrial fibrillation, renal disease, cancer, on lipid-lowering agent, and mean TC.

HR, hazard ratio; CI, confidence interval; CV, coefficient of variation; Q, quartile; SD, standard deviation; VIM, variability independent of the mean

Supplementary Table 3. The risk for the occurrence of venous thromboembolism according to the quartiles of total cholesterol variability in women

|  |  |  |  |  |  | Multivariable model (1) |  |  | Multivariable model (2) |  |  |
| --- | --- | --- | --- | --- | --- | --- | --- | --- | --- | --- | --- |
|  | Number of  participants | Number of  events | Event rate (%) (95% CI) | Person-years | Incidence rate (per 1000 person-years) | Adjusted HR (95% CI) | *P*-value | *P*-value for trend | Adjusted HR (95% CI) | *P*-value | *P*-value for trend |
| CV |  |  |  |  |  |  |  | <.001 |  |  | <.001 |
| Q1 | 62294 | 725 | 1.16  (1.08, 1.25) | 762786.58 | 0.95 | 1 (reference) |  |  | 1 (reference) |  |  |
| Q2 | 62295 | 678 | 1.09  (1.01, 1.17) | 764122.74 | 0.89 | 0.98  (0.88, 1.09) | 0.707 |  | 1.00  (0.90, 1.11) | 0.998 |  |
| Q3 | 62295 | 739 | 1.19  (1.1, 1.27) | 763558.28 | 0.97 | 1.02  (0.92,1.13) | 0.742 |  | 1.03  (0.95, 1.12) | 0.307 |  |
| Q4 | 62295 | 927 | 1.49  (1.39, 1.58) | 759651.75 | 1.22 | 1.06  (1.01, 1.11) | 0.002 |  | 1.06  (1.01, 1.12) | 0.004 |  |
| SD |  |  |  |  |  |  |  | <.001 |  |  | <.001 |
| Q1 | 62292 | 610 | 0.98  (0.9, 1.06) | 763727.77 | 0.8 | 1 (reference) |  |  | 1 (reference) |  |  |
| Q2 | 62287 | 685 | 1.1  (1.02, 1.18) | 764380.39 | 0.9 | 1.03  (0.97, 1.09) | 0.162 |  | 1.03  (0.98, 1.08) | 0.157 |  |
| Q3 | 62301 | 757 | 1.22  (1.13, 1.3) | 763197.89 | 0.99 | 1.06  (0.95, 1.18) | 0.308 |  | 1.05  (0.94, 1.17) | 0.384 |  |
| Q4 | 62299 | 1017 | 1.63  (1.53, 1.73) | 758813.3 | 1.34 | 1.07  (1.01, 1.13) | 0.003 |  | 1.07  (1.01, 1.12) | 0.004 |  |
| VIM |  |  | #DIV/0! |  |  |  |  | <.001 |  |  | <.001 |
| Q1 | 62294 | 610 | 0.98  (0.9, 1.06) | 763752.48 | 0.80 | 1 (reference) |  |  | 1 (reference) |  |  |
| Q2 | 62295 | 685 | 1.1  (1.02, 1.18) | 764478.94 | 0.90 | 1.03  (0.97, 1.09) | 0.163 |  | 1.04  (0.97, 1.10) | 0.196 |  |
| Q3 | 62295 | 757 | 1.22  (1.13, 1.3) | 763124.06 | 0.99 | 1.06  (0.95, 1.18) | 0.305 |  | 1.05  (0.94, 1.17) | 0.378 |  |
| Q4 | 62295 | 1017 | 1.63  (1.53, 1.73) | 758763.88 | 1.34 | 1.08  (1.01, 1.15) | 0.009 |  | 1.07  (1.01, 1.12) | 0.012 |  |

Multivariable model (1) was adjusted for age, sex, body mass index, household income levels, smoking, alcohol consumption, regular physical activity, hypertension, diabetes mellitus, dyslipidemia, stroke, atrial fibrillation, renal disease, cancer, and on lipid-lowering agent.

Multivariable model (2) was adjusted for age, sex, body mass index, household income levels, smoking, alcohol consumption, regular physical activity, hypertension, diabetes mellitus, dyslipidemia, stroke, atrial fibrillation, renal disease, cancer, on lipid-lowering agent, and mean TC.

HR, hazard ratio; CI, confidence interval; CV, coefficient of variation; Q, quartile; SD, standard deviation; VIM, variability independent of the mean

**Comment)** CV, SD, VIM are collinear and based on the same parameters (mean, SD). It leads to the same risk estimates for each parameter. Could this be discussed?

**Answer)** Recently, the variability of parameters has been emerged as new biomarkers for cardiovascular disease for overcoming the low reliability and reproducibility of spot biomarker measurement. In various articles about the association of variability of biomarker and cardiovascular disease, three calculation methods including SD (standard deviation), CV (coefficient of variation), and VIM (variability of independent of mean) have been widely used. First, SD is calculated as the square root of average squared deviation from the mean and a high SD indicates that data points are more spread out from the mean and that indicates high variability. However, SD could be increases as the mean increases, thus this phenomenon was controlled by dividing SD by the mean, resulting in a value that excludes the influence of the mean. This value is known as the CV. The lower CV indicates lower variability. However, there still have been concerns that CV is not totally independent from the influence of mean value. As a result, VIM has been widely used. VIM is not affected by the mean of dataset and calculated by dividing the SD by the mean to the power of a certain exponent. VIM has a strength to compare the variability with different means.

Therefore, CV overcomes the limitation of SD, while VIM also provide a way to overcome the limitation of CV. Thus, all of these parameters seemed to be essential for analyzing the variability of parameter. In our analysis, all the parameters about total cholesterol variability showed consistent positive trends to the development of venous thromboembolism (VTE). This finding is strengthening the result that the higher association between TC variability and VTE development.

**Comment)** The article needs editing throughout: **i)** First, I suggest using “VTE” instead of “VT” for venous thromboembolism, as it is a more common abbreviation. Also, please use “DVT” for deep vein thrombosis and “PE for pulmonary embolism. **ii)** The manuscript is sometimes unclear or imprecise, particularly the introduction (the first sentence is hard to understand, second paragraph on risk factors should be edited (major risk factors for VTE are surgery, immobilization/fracture of lower limbs, cancer, APS)); all other risk factors are at intermediate risk for VTE; I would tone down the association between atrial fibrillation, renal insufficiency and VTE as well in paragraph. **iii)** Considering putting all criteria for definition of cardiovascular risk factors and disease in an appendix instead of listing all ICD codes, etc. **iv)** Line 274 to 296 should be rephrased. **v)** Line 290 to 293: it is not clear whether the authors refer to their papers or others.

**Answer)** Thank the reviewer’s kind suggestion.

i) According to the reviewer’s suggestion, we used VTE instead of VT for venous thromboembolism, and DVT for deep vein thrombosis and PE for pulmonary embolism through entire manuscript. Thus, the title of our manuscript was changed from “Association of total cholesterol variability with risk of venous thrombosis: a nationwide cohort study” to “Association of total cholesterol variability with risk of venous thromboembolisms: a nationwide cohort study”.
ii) Thank reviewer for this valuable comment. The organization of paragraphs in introduction was modified. Thus, the first sentence of our manuscript was revised. In addition, the description about the intermediate risk factors for VT was also toned down.

Page 5 line 58-62

*“B-type natriuretic peptide, troponin, and C-reactive protein and serum lipid profiles have known to be important biomarkers for cardiovascular (CV) events [1-2]. The level of biomarkers in most biomarker studies has been determined through spot measurements, mainly because of cost constraints and the timing of blood sampling. However, this method was accompanied by reliability and reproducibility problems [1,3-4]”*

Page 5 line 68-70

*“Cancer, antiphospholipid syndrome (APS), fracture, obesity, renal failure and chronic inflammatory disease are known risk factors for the development of VTE [7]”*

Page 5 line 72-75

*“Other studies have found a relationship between increased TC variability and end-stage renal disease and atrial fibrillation (AF) that are known to have relationship with the development of VTE [9-10]”*

iii) To achieve a concise composition of our manuscript, all criteria for various cardiovascular risk factors and ICD-10 codes were moved to supplemental table 1 instead of method section in main manuscript.

Page 7 line 103-104

*“The detailed definition of various covariates in our study was described in S1 Table.”*

iv) We agree that the paragraph has awkward construction with inaccurate English expression. Thus, we modified the whole paragraph that reviewer pointed out.

Page 12 line 227-Page 13 line 247

*“VTE is a significant global health issue, with several risk factors associated with its development, including malignancy, older age, immobilization, obesity, recent fracture, surgery and hematologic heritable disorders [7]. In addition, both idiopathic and secondary VTE are known to have coexisting risk factors for cardiovascular diseases such as HET, DM, smoking and dyslipidemia [18].*

*Dyslipidemia, in particular, has been postulated to contribute to the development of VTE due to the influence of lipids and lipoproteins on hemostasis by modulating the expression and function of procoagulants, fibrinolytic, and theological factors [19]. However, studies investigating the association between lipid profiles and the development of VTE have yielded inconsistent results, with most studies relying only on spot measurement of lipid levels [20].*

*However, our study utilized TC variability, which is a more reliable measure than spot measurements. We found that TC variability had a positive impact on the development of VTE, regardless of different parameters for TC variability, including CV, SD and VIM. Furthermore, in subgroup analyses, TC variability consistently showed a positive impact on the development of various type of VTE, including DVT and PE, which have high mortality and recurrence rates [21]. Our finding suggested that assessing TC variability may be important for patients at high risk of VTE, such as those with malignancy, obesity, immobilization, and the use of oral contraceptives [7]. This caution could help to prevent fatal PE or DVT.*

v) We are very sorry to unclear and vague expression in our manuscript. We reconstructed the sentences and paragraph that pointed out to align with the content and overall flow of our manuscript.

Page 14 line 265-270

*“Furthermore, in subgroup analyses, TC variability consistently showed a positive impact on the development of various type of VTE, including DVT and PE, which have high mortality and recurrence rates [21]. Our finding suggested that assessing TC variability may be important for patients at high risk of VTE, such as those with malignancy, obesity, immobilization, and the use of oral contraceptives [7]. This caution could help to prevent fatal PE or DVT.”*

Thank the reviewer’s kind comment again.
